# The Geographic Spread of the CCR5 Δ32 HIV-Resistance Allele

**DOI:** 10.1371/journal.pbio.0030339

**Published:** 2005-10-18

**Authors:** John Novembre, Alison P Galvani, Montgomery Slatkin

**Affiliations:** **1**Department of Integrative Biology, University of California, Berkeley, California, United States of America; Cornell UniversityUnited States of America

## Abstract

The Δ32 mutation at the CCR5 locus is a well-studied example of natural selection acting in humans. The mutation is found principally in Europe and western Asia, with higher frequencies generally in the north. Homozygous carriers of the Δ32 mutation are resistant to HIV-1 infection because the mutation prevents functional expression of the CCR5 chemokine receptor normally used by HIV-1 to enter CD4+ T cells. HIV has emerged only recently, but population genetic data strongly suggest Δ32 has been under intense selection for much of its evolutionary history. To understand how selection and dispersal have interacted during the history of the Δ32 allele, we implemented a spatially explicit model of the spread of Δ32. The model includes the effects of sampling, which we show can give rise to local peaks in observed allele frequencies. In addition, we show that with modest gradients in selection intensity, the origin of the Δ32 allele may be relatively far from the current areas of highest allele frequency. The geographic distribution of the Δ32 allele is consistent with previous reports of a strong selective advantage (>10%) for Δ32 carriers and of dispersal over relatively long distances (>100 km/generation). When selection is assumed to be uniform across Europe and western Asia, we find support for a northern European origin and long-range dispersal consistent with the Viking-mediated dispersal of Δ32 proposed by G. Lucotte and G. Mercier. However, when we allow for gradients in selection intensity, we estimate the origin to be outside of northern Europe and selection intensities to be strongest in the northwest. Our results describe the evolutionary history of the Δ32 allele and establish a general methodology for studying the geographic distribution of selected alleles.

## Abstract 2

A spatially explicit model of the Δ32 mutation, which confers resistance to HIV-1 infection, reveals its spread across Europe and provides a general method for tracking the geographic spread of selected alleles.

## Introduction

The geographic spread of advantageous alleles is fundamental to evolutionary processes, including the geographic distribution of adaptive traits, the cohesiveness of species, and the spatial dynamics of coevolution between pathogens and their hosts. Various theoretical models describe the dynamics of how advantageous alleles spread within a population, but few well-studied examples exist, particularly in humans, of how advantageous alleles spread geographically.

The CCR5 Δ32 mutation is a good example of an advantageous allele with a well-characterized geographic distribution. The Δ32 mutation currently plays an important role in HIV resistance because heterozygous carriers have reduced susceptibility to infection and delayed onset of AIDS, while homozygous carriers are resistant to HIV infection [1]. The mutation is found principally in Europe and western Asia, where average frequencies are approximately 10%, although the frequency varies within this geographic area. HIV only recently emerged as a human pathogen, so researchers were surprised when various sources of evidence showed strong selection in favor of Δ32 throughout its history. The age of the Δ32 allele has been estimated to be between 700 and 3,500 y based on linkage disequilibrium data [2,3], and recent ancient DNA evidence suggests the allele is at least 2,900 y old [4]. If Δ32 were neutral, population genetics theory predicts it would have to be much older given its frequency. The alternative explanation is that the Δ32 mutation occurred recently and then increased rapidly in frequency because of a strong selective advantage [2,5]. Quantitative studies have concluded that heterozygous carriers of Δ32 in the past had a fitness advantage of at least 5% and possibly as high as 35% [2,3]. Bubonic plague was initially proposed as the selective agent [2], but subsequent analysis suggested that a disease like smallpox is a more plausible candidate ([6–8], with reviews in [9–11]).

**Figure 1:**
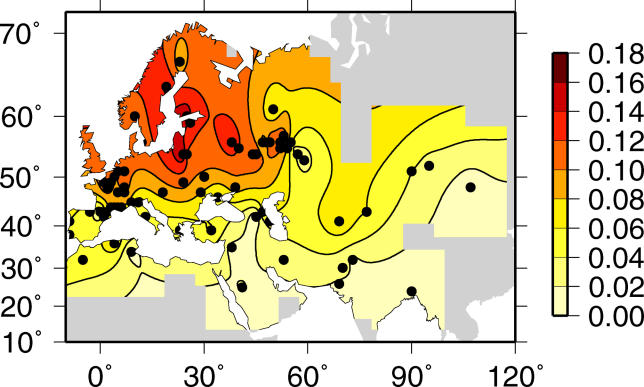
Shaded Contour Map of Δ32 Allele Frequency Data The sampling locations are marked by black points. The interpolation is masked in regions where data are unavailable.

**Figure 2:**
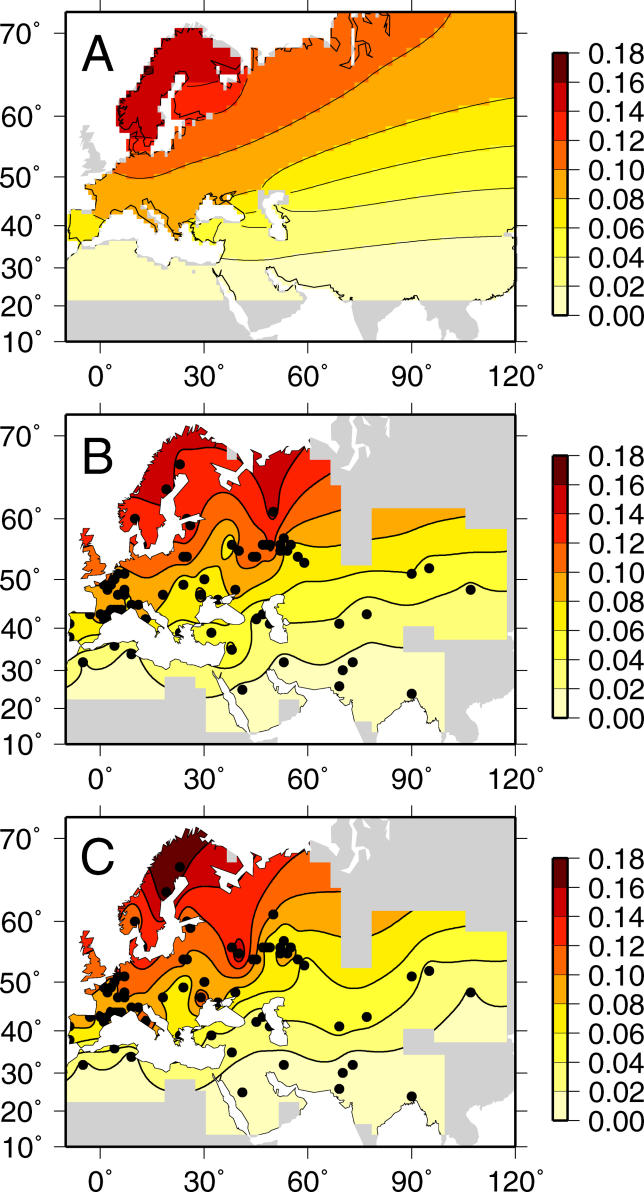
An Example of the Allele Frequency Surface and Simulated Data (A) The underlying allele frequency surface generated by the PDE model using MLEs for the parameters. The coarseness of the surface and irregular coastlines are due to the resolution of the simulated habitat (see Figure S1). (B and C) Two replicates of simulated data obtained using the same sampling locales and sample sizes as in the dataset and displayed using the same interpolation methods and contours as in Figure 1. The results show that underlying smooth, unimodal allele frequency surfaces can give rise to irregular, multimodal observed allele frequency surfaces.

To understand the origin and spread of Δ32, we modeled the effects of selection and dispersal on the allele. The Δ32 mutation is found only in European, West Asian, and North African populations. The allele frequency exhibits a north–south cline with frequencies ranging from 16% in northern Europe to 6% in Italy and 4% in Greece (Figure 1; [2,5,12–17]). The broadest area of high frequency is located in northeastern Europe, particularly the Baltic region, as represented by samples from Sweden, Finland, Belarus, Estonia, and Lithuania. There are additional peaks of frequency in samples from the northern coast of France, the Russian cities of Moscow and Ryazan, and portions of the Volga–Ural region of Russia. Ashkenazi Jews have high frequencies of Δ32, but this is likely due to founder effects unique to their history rather than the general process of dispersal that spread the allele in other populations [18].

Previous discussion of the geographic distribution of Δ32 has focused on the north–south cline in frequency. Lucotte and Mercier [12] suggested that the cline and other features of the geographic distribution imply a Viking origin. In particular they proposed that the allele was present in Scandinavia before 1,000 to 1,200 y ago and then was carried by Vikings northward to Iceland, eastward to Russia, and southward to central and southern Europe. The age and geographic distribution of the allele are consistent with the qualitative predictions of the Viking hypothesis [12,17], but there has been no quantitative analysis of the Viking hypothesis or alternative hypotheses.

One alternative is that a northern origin coupled with typical levels of dispersal in Europe is adequate to explain the geographic distribution of Δ32. Under this hypothesis, rare long-distance dispersal events, such as Viking dispersal, play a minor role in the spread of the advantageous allele. Another alternative is that the allele may have arisen in central Europe and increased to a higher frequency in the north because of a geographical gradient in selection intensity [19]. There are two plausible biological causes for a gradient in selection. First, the selective advantage of Δ32 may have been larger in the north. This hypothesis stems from anecdotal evidence that indicates smallpox epidemics were more intense in northern Europe [20]. A second mechanism is that a selective cost associated with the Δ32 allele may have been stronger in the south, and thus the overall selection intensity in favor of Δ32 may have been weaker in the south and stronger in the north. While there is little direct evidence that Δ32 carriers are more susceptible to general infection [10,11], the plausibility of a selective cost of Δ32 is supported by evidence that chemokines play an important role in inflammatory responses to infection [21,22] and by studies with mice that show that CCR5 knockouts have poor immune responses to various pathogenic infections [23–25]. These results suggest some pathogens may have an advantage infecting Δ32 carriers because the immune response is impaired by the absence of functional CCR5 chemokine receptors. If such pathogens tended to be more prevalent in the temperate climates of southern Europe, then a selection gradient would arise. It is even plausible that Δ32 could be disadvantageous in certain areas where the protective effect is outweighed by the disadvantage of a weakened immune response. In a model with selection gradients, Viking dispersal may still contribute to the spread of the allele, but the geographic origin of the allele and the influence of spatially variable selection differ from that in the Viking hypothesis.

A further question regarding the geographic spread of Δ32 is whether the historical selective agent acted only in Europe and western Asia or on a larger geographic scale. In the former case, the restriction of Δ32 to Europe and western Asia is explained by spatially varying selection, and in the latter, by insufficient time for the allele to have dispersed farther.

Here we fit a simple population genetic model to the geographic distribution of Δ32 in order to infer features of the processes of dispersal and selection that shaped the historical spread of the allele. In particular we conclude that given current estimates of the age of the Δ32 allele, the allele must have spread rapidly via long-range dispersal and intense selection to attain its current range. We find the Δ32 allele is likely restricted geographically because of limited time to disperse rather than local selection pressures. In addition, we show that the data are consistent with origins of the mutation outside of northern Europe and modest gradients in selection.

## Results

To examine the geographic distribution of Δ32, we adapted Fisher's deterministic “wave of advance” [26] model of selection and dispersal to a geographically explicit representation of Europe and western Asia. The wave-of-advance model is a continuous-time, continuous-space, partial-differential-equation (PDE) model that describes the change in allele frequency at any point in the range in terms of the effects of dispersal and selection. The model treats dispersal as a diffusion process, which implicitly assumes the effects of dispersal can be approximated by considering only the mean and variance of the dispersal distribution. Furthermore, dispersal is assumed to be homogeneous across the range and isotropic (i.e., the mean of the dispersal distribution is zero). In the simplest form of the model, the allele under selection is additive in effect and selection intensity is assumed to be homogeneous across the range. We then extended the model to allow for both east–west and north–south gradients in selection intensity. The parameters of the model are the initial population density *(D),* the initial position of the mutation in terms of latitude and longitude (*x*_0_ and *y*_0_, respectively), and the ratio *(R)* of the variance in the parent–offspring dispersal distance distribution (σ^2^) to the additive selection coefficient *s*. When a gradient in selection intensity was incorporated, *s_c_* was the selection coefficient in the center of the gradient and *G_NS_* and *G_EW_* were the percent changes in the selection coefficient per kilometer of north–south and east–west distance, respectively.

To apply the model to allele frequency data sampled from different locations, we combined the spatially explicit PDE model with a binomial sampling scheme (see Materials and Methods). With this approach the data are viewed as being a set of binomial samples from an underlying, unobserved allele frequency surface that is generated by the PDE model. One feature of this model is that while the underlying allele frequency surface produced by the PDE model may be unimodal and smooth, the resulting data are expected to be noisy and multimodal owing to the binomial sampling step. The multimodal nature of data expected under the model approximates that found in the dataset of Δ32 allele frequencies we collated from 71 locations across Europe, northern Africa, and Asia (Figure 1; data described in Materials and Methods). Indeed when simulations are used to generate allele frequency data with identical sampling locations and sample sizes as in the collated dataset, multiple peaks are often found that are similar to those observed in real data (Figure 2). This result demonstrates that specific regions of high frequency are not necessarily generated by unusual local conditions or specific migration events, but can arise because of sampling in models with homogeneous dispersal.

To estimate the parameters of the model, we derived a likelihood function based on binomial sampling from the deterministic allele frequency surface. Estimating parameters via maximum likelihood requires an optimization step, and here we use a simple grid search. We found in applications to simulated data that the likelihood method with a grid search is able to estimate parameters with reasonable accuracy (Table 1).

**Table 1:**
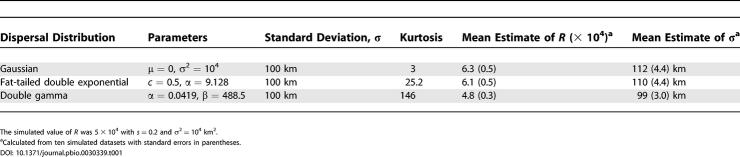
The Effect of the Dispersal Distribution on the Estimates of *R* and σ

**Figure 3:**
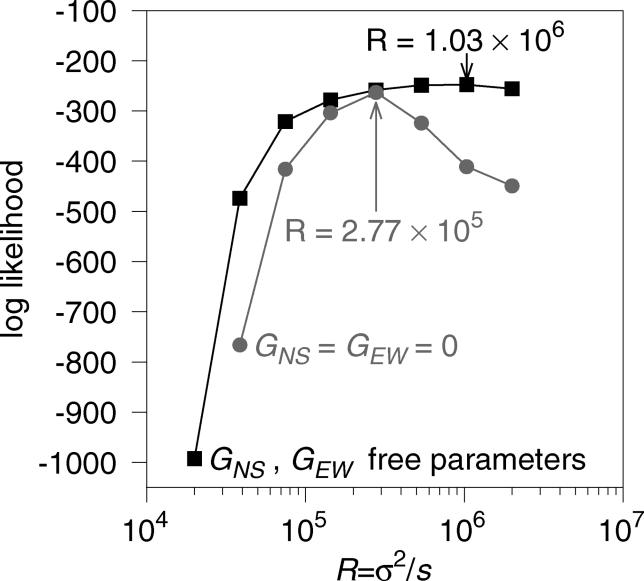
Profile Likelihood for *R* The grey line shows the log profile likelihood for *R* when selection is assumed to be uniform spatially (*G_NS_* = *G_EW_* = 0). The MLE of *R* in this case is 2.77 × 10^5^ with a log-likelihood of −263.0. The black line shows the profile likelihood when selection gradients are incorporated into the model (*G_NS_* and *G_EW_* are free parameters). The corresponding MLE of *R* is 1.03 × 10^6^ with a log likelihood of −247.7.

**Figure 4:**
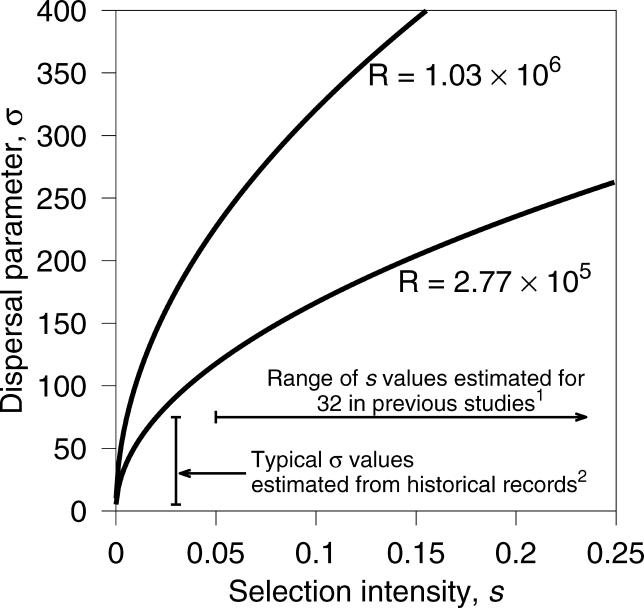
The Dispersal Parameter σ as a Function of the Selection Intensity *s* The curves are drawn for the two MLEs of *R* and labeled accordingly. ^1^Based on estimates in [2] and [3]. ^2^From Table 1 of [27].

The result of the maximum likelihood estimation is that values of *R =* σ^2^/*s* on the order of 10^5^ and 10^6^ km^2^ have the highest likelihood. The maximum likelihood estimate (MLE) of *R* depends on whether selection gradients are allowed. When *G_NS_* = *G_EW_* = 0, the estimate of *R* is 2.77 × 10^5^ km^2^ with the profile likelihood falling off nearly symmetrically for higher and lower values (Figure 3). When *G_NS_* and *G_EW_* are treated as free parameters, the MLE of *R* is 1.03 × 10^6^ km^2^ with a steep drop in likelihood for values less than 10^5^ and a gradual decline for values greater than 10^6^ (Figure 3). Values of *R* on the order of 10^4^ km^2^ or smaller result in an expected geographic distribution of Δ32 that is too restricted to fit the data, and values of *R* on the order of 10^7^ result in a distribution that is far too broad. Figure 4 demonstrates the values of σ and *s* implied by these estimates. The value of σ is consistently larger than 100 km for *s* ≥ 0.05. These values of σ are larger than estimates based on studies of historical and modern dispersal, which indicate that σ ranges from 1 to 75 km in European populations [27]. Conversely, values of σ < 75 km, imply values of *s ≤* 0.02, which are lower than estimates of *s* ≍ 0.05–0.35 obtained in previous studies based on Δ32 frequency and linkage disequilibrium data [2,3].

The time required in the model to reach a frequency of 16% for a fixed value of *s* is indicative of the age of the Δ32 allele. Using this information we find that strong selection (*s* ≥ 0.1) is necessary to reconcile the spatially explicit model of Δ32 with estimates of the allele age. The linkage disequilibrium data of Stephens et al. [2] and Libert et al. [5] suggest the age of the Δ32 allele falls in the range of 700–3,500 y, and recent ancient DNA data suggest the allele arose at least 2,900 y ago[4]. Assuming *s* = 0.1, we found across the range of origins we investigated that 130–156 generations (3,250–3,900 y) are required to reach current frequencies, which is consistent with the upper range of allele age estimates from linkage disequilibrium data and the ancient DNA data. To generate younger allele ages would require selection coefficients larger than 0.1. For weaker selection coefficients, the ages must be much larger. As an example, 650–780 generations (16,250–19,500 y) are required to reach current frequencies assuming *s* = 0.02.

We next investigated whether the data reject the hypothesis of uniform selection gradients. We used a likelihood ratio test that compares the maximum likelihood achieved when *G_NS_* and *G_EW_* are both restricted to zero to the maximum likelihood obtained when *G_NS_* and *G_EW_* are estimated from the data. As shown in Figure 3, when *G_NS_* and *G_EW_* are free parameters, the maximum log-likelihood is approximately −248. With *G_NS_* and *G_EW_* fixed to zero, we find the maximum likelihood at −263. As a result, the likelihood ratio statistic is strongly significant (likelihood ratio statistic = 30, df = 2, *p <* 10^−5^), such that we can reject the null hypothesis of uniform selection.

**Figure 5:**
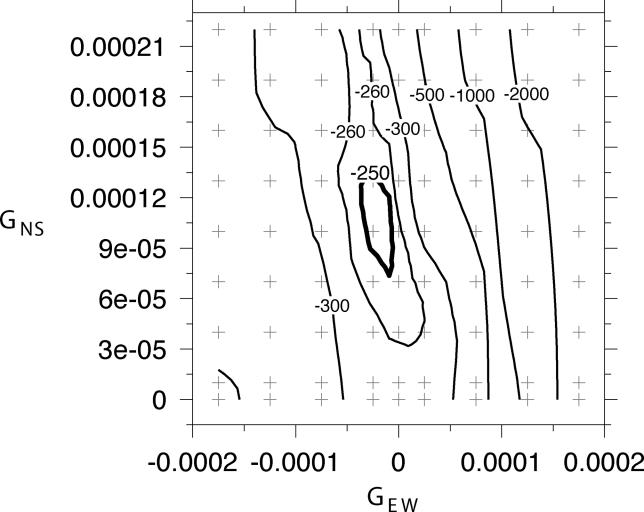
Profile Likelihood Surface for *G_NS_* and *G_EW_* The plus signs indicate locations where the likelihood was evaluated. The dark contour at −250 marks the −2 log-likelihood support region for the estimates of *G_NS_* and *G_EW_*.

In the model with selection gradients, selection was inferred to be stronger in the north and in the west, with the north–south selection gradient being steeper than the east–west selection gradient. In particular, the estimates of *G_NS_* and *G_EW_* were *G_NS_* = 1.3 × 10^−4^ and *G_EW_* = −0.25 × 10^−4^. The profile likelihood surface for *G_EW_* and *G_NS_* shows a peak such that the likelihood drops off steeply from the maximum in both directions along the *G_EW_* axis while only declining gradually from the maximum in the *G_NS_* axis (Figure 5). The magnitude of the gradients is not extreme. For instance, the gradient of *G_NS_* = 1.3 × 10^−4^ km^−1^ implies a selection intensity at the latitude of Oslo that is a 21% increase on the selection intensity at the latitude of Milan (e.g., *s =* 0.23 in Oslo versus *s =* 0.19 in Milan). Similarly, *G_EW_* = −0.25 × 10^−4^ km^−1^ generates a selection intensity in Copenhagen that is 5% greater than in Moscow (e.g., *s =* 0.22 in Copenhagen and *s =* 0.21 in Moscow).

**Figure 6:**
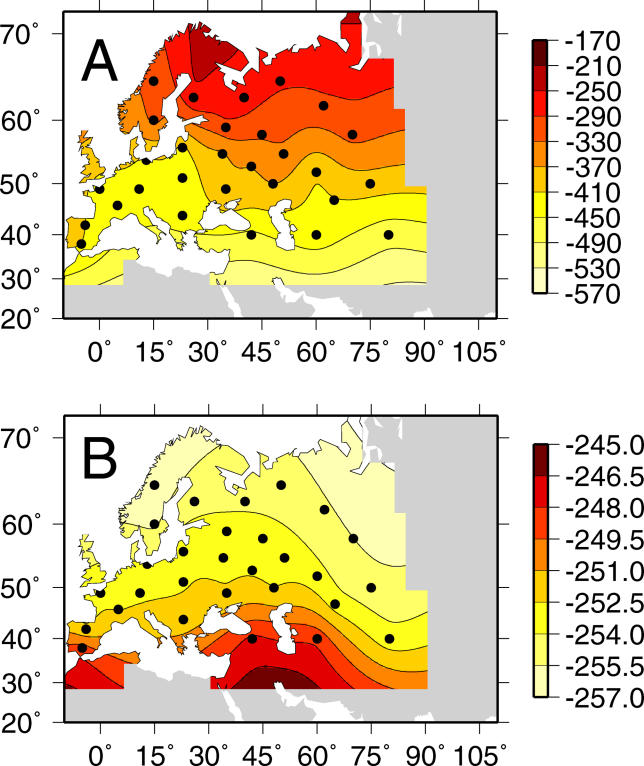
Likelihood Surfaces for the Origin of the Allele (A) Assuming selection intensity is uniform spatially (i.e., *G_NS_* = *G_EW_* **=** 0). (B) Allowing for north–south and east–west spatial gradients in selection (i.e., *G_NS_* and *G_EW_* are free parameters). Likelihoods were calculated at each of the black points and the surface was obtained by interpolation.

Regarding the geographic origin of Δ32, we found that if selection is constrained to be spatially uniform, the origin is localized to a region east of the Baltic (Figure 6A; parameter set NE in Table 2). Moreover, the likelihood surface drops off dramatically outside of this region. If gradients in selection are incorporated, the origins with the highest likelihood are in southern Europe, with the maximum likelihood origin west of the Caspian (Figure 6B; parameter set C in Table 2). An origin in Spain (parameter set SW in Table 2) and an origin east of the Caspian (parameter set SE in Table 2) are within two log-likelihood units of the maximum. All three origins are coupled with an inference of a south-to-north and east-to-west increase in selection intensity. Origins in northern Europe have low likelihood, although the likelihood surface is fairly flat so that origins with a latitude as high as 58° are within six log-likelihood units of the maximum.

**Table 2:**
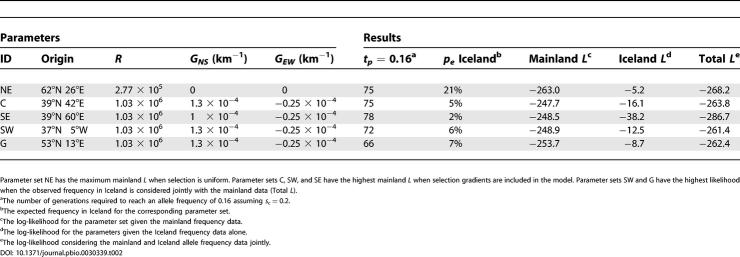
Detailed Results for Parameter Sets with High Likelihoods

The underlying allele frequency surface generated by the MLE parameters is a qualitative indicator of the goodness of fit of the model (see Figure 2A). The frequency surface is broadly consistent with the observed allele frequency distribution (see Figure 1), although the estimated underlying distribution shows allele frequencies to be higher than observed in the northernmost part of Scandinavia and in parts of southern Europe. In addition, the estimated surface shows a region in the northeast of Russia where no data points exist and Δ32 is predicted to be present in frequencies of 8%–10%. Simulated data from the MLE allele frequency surface, with sample sizes and locations that are identical to those in the original dataset, have features such as multiple peaks that are present in the original data (for two examples of simulated data see Figure 2B and 2C). When we quantitatively tested the goodness of fit using a *G*-test, we rejected the null hypothesis that the data are derived from the model (*G* = 205.6, df = 66, *p* < 10^−5^). We also found that the data are overdispersed relative to the variance expected under a binomial distribution. The overdispersion parameter φ (see Materials and Methods) was estimated to be 3.1 where a value of one is expected under the model.

Finally, to better understand the history of Δ32, we considered an extension of the model that included the dispersal of the allele to Iceland. Iceland was colonized principally from Scandinavia at approximately 900 CE [28] and present-day Iceland samples show a high frequency of Δ32 [13]. To model the frequency of Δ32 in Iceland, we used a single-population selection model for Iceland with initial conditions based on the mainland PDE model (see Materials and Methods). We used this simple model to calculate an expected frequency of Δ32 in Iceland at the present day. Data from present-day Iceland show a frequency of 14.7% in a sample of 204 chromosomes [13]. Hypothetical origins outside of northern Europe result in underestimates of the frequency in Iceland, and a northern origin results in a slight overestimate (for examples, see Table 2). To integrate the Iceland results into the analysis of the mainland frequency data, we used the fact that the observation in Iceland is an independent data point and added the log-likelihood of the Iceland data to that obtained from the mainland allele frequency data. An origin in Spain (parameter set SW in Table 2) is found to be the MLE with a log-likelihood of −261.0. We also found that an origin at 53°N 13°E in northern Germany (parameter set G in Table 2) has a log-likelihood of −262.4 and is the only other origin within two log-likelihood units of parameter set SW. Thus, including historical information about Iceland shifts the estimated origin to western Europe, with likely origins in Spain or northern Germany.

## Discussion

We can draw several conclusions from our analysis of the geographic distribution of the Δ32 allele. First, the results suggest that strong selection (*s ≥* 0.10) and long-distance dispersal of humans (σ > 100 km) are necessary to explain the current geographic distribution of the Δ32 allele. Values of σ > 100 km are larger than the estimates of 1–75 km found in various studies using historical records for Europeans [27]. If, however, the Δ32 allele is older and less advantageous than previously estimated [3,4,6], which has been suggested recently by [29], then our analysis of the geographic distribution becomes more consistent with previously published estimates of dispersal distances in European populations. Because our results depend on the ratio *R* = σ^2^/*s,* a smaller *s* implies estimates of σ that are closer to those based on historical records (see Figure 4), although still somewhat larger. For example, for our MLE of *R* = 1.03 × 10^6^, a value of *s* = 0.0075 corresponds to σ = 88 km. One explanation for the discrepancy between genetic and historical estimates of dispersal distance is that the estimates based on historical records may be too low because they do not reflect longer-distance dispersal events such as those associated with trade routes or population movements. Our larger estimate of σ may reflect such long-distance dispersal events.

Second, we conclude that if selection is spatially uniform, Δ32 arose by mutation in northeast Europe as suggested by Libert et al. [5]. This hypothesis is parsimonious because it does not require gradients in selection intensity. If selection is not spatially uniform, we find the geographic origin could be far from locations where Δ32 is currently in high frequency. We reach this conclusion because, in our model, dispersal dominates initially and spreads the new allele over a large geographic area before selection can increase the allele frequency locally. When we allow for selection gradients and take account of the data from Iceland, we conclude that Δ32 most likely originated either in Spain or northern Germany. The gradients in selection intensity needed are not extreme and are on the order of only a 20% relative difference between southern and northern Europe and a 5% relative difference between eastern and western Europe. Although allowing for selection gradients is less parsimonious, the model with selection gradients had a significantly higher likelihood than the model with uniform selection. The north–south gradient detected here is consistent with anecdotal evidence that smallpox was more prevalent in the north [20].

Third, our results show that the geographically restricted distribution of Δ32 is a result of Δ32 not having had time to disperse more widely, rather than resulting from a geographic restriction of selection favoring it. Given more time and no change in selection affecting Δ32, the allele would have spread over a wider area.

Our large estimates of dispersal are consistent with the Viking hypothesis of Lucotte and Mercier [12]. Moreover, when selection is assumed to be spatially uniform, the maximum likelihood origin is in southern Finland. However, incorporating gradients in selection provided significantly better fits to the data, and in models with gradients, origins in Scandinavia did not have high likelihoods. Thus, our likelihood-based analysis provides some support for the Viking hypothesis in that we detect a strong signature of long-range dispersal events, but it also raises the possibility that the allele arose outside of Scandinavia and spread into the region via dispersers from the south.

Our analysis makes a number of simplifying assumptions. Our model does not incorporate genetic drift. To examine the effect of ignoring drift, we simulated a stepping stone model with local deme sizes of *N_e_* = 2,500 and a selection coefficient of *s* = 0.05. We found that with drift, the allele frequency surface becomes somewhat more jagged than without drift, but the underlying shapes are still the same (results not shown). Concluding that drift is negligible based on the simulation results is conservative as the selection coefficient of Δ32 is most likely at least 0.05 [2,3] and the effective population size of a deme (where 1° latitude by 1° longitude may be considered a deme) would be greater than 2,500 even assuming 2000 BCE population densities (2.5 km^−2^; [30]) and *N_e_* as one-quarter of the census population size. Additional support for the idea that drift is unimportant for the large-scale patterns in this type of model comes from the analytical results of Kot et al. [31] for a similar model of branching random walks. They show that the average rate of expansion in a stochastic model is similar to that in a deterministic model.

Another assumption of our approach is that the allele under selection has an additive effect. We tested the robustness of our results to deviations from additivity by generating allele frequency surfaces in which the fitness advantage of Δ32 heterozygotes is kept constant and a range of fitness advantages of the Δ32/Δ32 genotype was assumed. We found that varying the degree of dominance had little effect on the geographic distribution of Δ32 (results not shown). The negligible importance of the fitness advantage of Δ32 homozygotes arises because the proportion of Δ32 homozygotes is sufficiently small throughout the history of Δ32 that the assumption regarding the fitness of the homozygote only has a minor effect.

Our use of diffusion equations assumes that only the mean and variance of the dispersal distribution are needed to model the effects of dispersal and that higher central moments such as kurtosis are negligible. Studies of Fisher's wave of advance in the ecology literature have shown that if kurtosis is non-negligible, as in the case of “fat-tailed” dispersal distributions (distributions whose tails are not exponentially bounded), the asymptotic behavior of the wave of advance changes so that the speed of the wave continually accelerates [32,33]. Because observed dispersal distributions for humans have been found to be leptokurtic (i.e., large kurtosis) [34], we considered the effect of a leptokurtic dispersal distribution on our results. We simulated the spread of an advantageous allele in a two-dimensional stepping stone model on a torus using three different dispersal distributions with varying degrees of kurtosis: a normal distribution, a double gamma distribution used by Cavalli-Sforza et al. [34] to fit human dispersal data, and a modified double exponential distribution used by Clark et al. [35] to describe fat-tailed seed dispersal data (see Table 1). These distributions were scaled to have a standard deviation of 100 km. We sampled data from the resulting spatial distributions of allele frequency, and estimated the ratio of dispersal to selection using the same method we applied to the Δ32 data. The results (Table 1) show that the effect of kurtosis on the estimates is very small: between all three distributions the estimates of *R* vary in a narrow range and the corresponding values of σ vary only between 98 and 110 km.

While violations of each of these simplifying assumptions (no genetic drift, additivity of the selective effect, a diffusion approximation for dispersal) are unlikely to have important effects on our estimates, the variance introduced by violations may contribute to the overdispersion observed in the data and the significant *G*-test statistic we computed. Another likely cause of the unexplained variance is that our model does not explicitly incorporate specific historical events. Information about particularly important dispersal events will help refine quantitative models of the evolution of Δ32. However, a challenge to developing such models is the difficulty of keeping them from becoming too parameter-rich or overburdened with assumptions regarding historical demographic events [36].

In summary, we present an approach to analyzing the geographic distribution of a selected allele. The approach allows us to estimate the ratio of dispersal to selection as well as fit gradients in selection to the observed allele frequency data. Our analysis confirms Δ32 has been under strong selection, and furthermore shows that long-range dispersal and selection gradients have been important processes in determining the spread of this advantageous allele. The results provide an insight into the history of Δ32 and into the processes that affect the geographic spread of advantageous alleles in humans.

## Materials and Methods

### Landmass data

We focused our analysis on the region extending from 22°N to 75°N and 10°W to 154°E. Topographic data were obtained from the ETOPO5 data assembled by the National Geophysical Data Center. The exact dataset used was a version with 1° latitude/longitude resolution that is provided as a standard dataset in MATLAB 7. The coastline data were extracted by taking all values above sea level to be land. A land bridge between Denmark and Sweden was added to model migration between the two closely separated land masses. An image of the habitat is available as Figure S1.

### Allele frequency data

A summary of Δ32 allele frequency data was constructed by pooling data from multiple published papers [12,14,19,37–43]. Latitude and longitude for 58 of the 71 samples were kindly provided by S. Limborska. For the remainder of samples, we used either the latitude and longitude of the city where the sample was collected or the latitude and longitude of a major city in the region sampled. We excluded any data points that were obtained from land masses not connected to the European mainland in our model, as well as allele frequency estimates from Ashkenazi Jews because of the unique founder effects in their history [18]. The allele frequency data are provided in Table S1.

### Model of selection and dispersal

To model the frequency of the Δ32 allele across Europe we used an approach based on Fisher's wave-of-advance theory [26]. The model is deterministic and based on a two-dimensional, nonlinear PDE. The PDE describes the function *p*(*x*,*y,t*), which represents the distribution of allele frequency across the *xy* plane at time *t:*



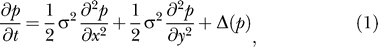



where Δ(*p*) is a nonlinear function of *p* that represents the change in allele frequency due to selection. The coefficient σ^2^ denotes the variance of the parent–offspring dispersal distance distribution. To calculate Δ(*p*), we assume fitnesses of 1 + *d,* 1 + *s,* and 1 for the Δ32/Δ32, Δ32/+, and +/+ genotypes, respectively. For this parameterization of selection, standard deterministic theory [44] gives the following result:



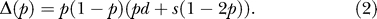



To incorporate gradients in selection, *s* and *d* are replaced with linear functions, denoted *s*(*x*,*y*) and *d*(*x*,*y*), respectively. In particular



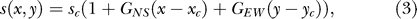



where *s_c_, x_c_,* and *y_c_* represent the selection coefficient and the *x* and *y* coordinates of the center of the habitat, respectively. This approach does not limit *s*(*x*,*y*) to being positive; that is, if the gradient in selection is strong enough, portions of the range may have negative selection coefficients. The results presented here are limited to the assumption of additivity, so *d*(*x*,*y*) = 2*s*(*x*,*y*), although the results are not sensitive to the assumption regarding *d* (see Discussion).

To represent the occurrence of the mutation at a single location in space with an initial local frequency of *p*_0_, we specified the initial conditions of the PDE solution to be



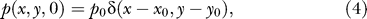



where δ(*x*,*y*) is a two-dimensional Dirac delta function, which takes on values close to one at *x* = 0 and *y* = 0, and values near zero for all other values of *x* and *y*. The value of *p*_0_ was calculated by the formula 1/*D,* where *D* is the initial population density. Population density only enters the model by determining the initial frequency. We generated results for *D* = 2.5 and *D* = 20. The two conditions correspond to published estimates of the population density in Europe at 1000 BCE and 1300 CE [30] and thus represent population density at the two orders of magnitude that are relevant for the origin of Δ32. The general results presented did not differ between *D* = 2.5 and *D* = 20, so we report results only for *D* = 2.5. For the boundary conditions, a model of reflecting boundaries was imposed. The assumption of reflecting boundaries is an implicit assumption that alleles are not lost or gained at the habitat boundaries.

For the application of the equations to a geographic habitat, we set the *x*-axis to be latitude and the *y*-axis to be longitude. In the results we report σ in units of kilometers. For rendering the habitat we work in coordinates of degrees latitude and longitude, so a simple conversion is needed to change σ in units of kilometers to units of degrees latitude and longitude. To convert from units of latitude we employ the number of kilometers per degree latitude as the scaling coefficient, which is a constant 111 km per degree latitude. To account for the decreasing amount of geographic distance represented by 1° longitude as one moves north, σ in the longitudinal axis is converted by taking σ/*m*(*x*) where *m*(*x*) is the number of kilometers per degree longitude at latitude *x*. Without this correction, there would be a nearly 4-fold difference in latitudinal dispersal between the lower edge (22°N) and upper edge (75°N) of the range we considered. A similar correction is applied to *s*(*x*,*y*), *d*(*x*,*y*), and *p*_0_.

### Numerical solutions

To solve the PDE for *p*(*x*,*y,t*), we used an alternating-direction implicit approach with Crank–Nicholson updates at each time step [45]. In this approach, the continuous habitat is discretized into elements of length Δ*x* and width Δ*y*. Time is discretized into segments of length Δ*t,* so that *p*(*x*,*y,t*) is represented by a three-dimensional matrix *P* ^(*n*)^ with elements *P^(n)^_j,k_* representing the value of *p*(*x*,*y,t*) at time *n*Δ*t* at a point (*j*Δ*x,k*Δ*y*) relative to the origin. Δ*x* and Δ*y* were set to 1° of latitude and longitude, respectively. For the results presented here Δ*t* was fixed at 0.005. Results were qualitatively similar for different values of Δ*x,* Δ*y,* and Δ*t,* provided that all three were sufficiently small. The accuracy of numerical solutions was confirmed by comparison to analytical results that exist for simple geometries and linear selection pressures (results not shown).

### Model for the frequency of Δ32 in Iceland

To model the frequency of Δ32 in Iceland, we used a standard single-population deterministic model of selection in which the additive selection intensity was set to *s* = 0.2 and the initial frequency was obtained from the PDE model. Specifically, the initial frequency was obtained by setting a selection Intensity of *s_c_* = 0.2 in the PDE model and recording the allele frequency at a representative location in Scandinavia (60°N 11°E) 44 generations (≍1,100 y) before the allele reaches 16% frequency. This approach neglects any possible founder effects associated with the founding of Iceland and any recurring migration between Iceland and mainland Europe. It also assumes the selection intensity in Iceland to be similar to that on the mainland.

### Maximum likelihood estimation

To obtain MLEs for the parameters of the model, we used a fixed allele age *t_a_* and supposed the number of Δ32 alleles observed at each sample locale arose as an independent binomial sample where the success probability at point (*x*,*y*) is determined by *p*(*x*,*y,t_a_*). The resulting likelihood function is



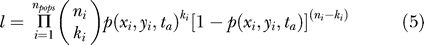



This likelihood approach benefits from taking into account the sample size at each sampling locale, so that the discrepancy between predicted and observed allele frequencies is penalized less at locations with smaller sample sizes. The value of *t_a_* used for the results was the time at which, given the other parameters, the maximum allele frequency first reached 16%, although using a value of 20% provided qualitatively similar results.

A grid-based method was used to produce a joint likelihood surface over *R, G_NS_, G_EW_, x*_0_, and *y*_0_. We used a grid for *R* that had eight values between 2 × 10^4^ and 2 × 10^6^ spaced evenly on a logarithmic scale; a grid for the geographic origins *x*_0_ and *y*_0_ that contained the 29 locations indicated by the points in Figure 6; a grid for *G_NS_* that started at 0 and then covered from 1 × 10^−5^ to 22 × 10^−5^ with increments of 3 × 10^−5^; and a grid for *G_EW_* that included zero as well as a range from −17.5 × 10^−5^ to 17.5 × 10^−5^ with increments of 5 × 10^−5^. The resulting grid contained 14,848 points in the five dimensions of *R, G_NS_, G_EW_, x*_0_, and *y*_0_.

### Evaluating the goodness of fit

To asses the goodness of fit of the model we performed a standard *G*-test. The *G*-test statistic can be formulated as 2(*L_n_ −L*_5_) where *L*_5_ is the log-likelihood computed using the MLE values in our full five-parameter diffusion-based model, and *L_n_* is the log-likelihood computed using the observed sample frequencies as the respective population frequencies for the binomial distributions in the likelihood function (equation 5). For our dataset, *L_n_* = −144.9 and *L*_5_ = −247.7. We also estimated the overdispersion parameter φ by the ratio of the *G*-test statistic to the number of degrees of freedom. Under the null hypothesis the estimate of the overdispersion parameter is expected to be one, and for large samples, values greater than one are indicative of overdispersion in the data.

### Evaluating the effect of kurtosis

To evaluate the effect of kurtosis we used simulations on a two-dimensional stepping stone habitat of 121 × 121 demes placed on a torus-shaped habitat arranged in a uniform distribution on (−6,000 km, 6,000 km) along the *x* and *y* axes. The origin of the allele was chosen to be at *x*_0_ = 0 and *y*_0_ = 0, and the initial frequency of the allele was set to 10^−5^. The simulations were stopped when the allele frequency became greater than 16%. Selection was incorporated with an additive allele with *s* = 0.2. Dispersal was modeled using three dispersal distributions. Here, for simplicity, we present the one-dimensional version of each dispersal kernel. The two-dimensional distribution was found by assuming dispersal along each axis was probabilistically independent and taking the products of the corresponding one-dimensional distributions. The first distribution was a mean-zero Gaussian distribution:



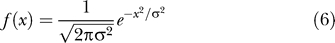



The second was a modified double exponential that when *c* < 1 is not exponentially bounded, and thus qualifies as a fat-tailed dispersal distribution:



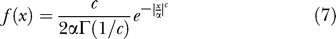



The third was a double gamma distribution that was used by Cavalli-Sforza et al. [34] to fit historical data on human dispersal:



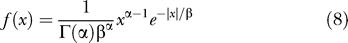



All three distributions were parameterized to have a standard deviation equal to 100 km, so that the effect of kurtosis alone could be assessed. For the shape parameter of the double gamma distribution we used the value of 0.0419 estimated by Cavalli-Sforza et al. [34] for human historical data. The resulting double gamma distribution had a kurtosis of 146.2. For the modified double exponential distribution, we used a shape parameter (*c* = 1/2) that gives reasonable kurtosis (*K* = 25.2) and guarantees the tails of the distribution are not exponentially bounded. The resulting allele frequency surfaces were binomially sampled at 49 evenly spaced locations with samples of size 120 to construct a simulated allele frequency dataset. The data were then passed to the likelihood-based method used on the Δ32 data but with *G_NS_, G_EW_, x*_0_, and *y*_0_ all fixed to zero, so that *R* was the only parameter to estimate. For the grid search we used a grid of *R* values with nine points from 3.3 × 10^4^ to 1 × 10^5^. The mean and standard error for estimates of *R* and σ reported in Table 1 are the average of ten replicates for each dispersal distribution.

## Supporting Information

Figure S1 The Geographic Area Used in the PDE Model The grey squares mark the geographic area used for numerical solution of the PDE. Coastlines are overlaid in the figure only for reference.
(38 KB PDF).

Table S1 Allele Frequency Data Used in the Analysis (33 KB PDF).

## Abbreviations

MLE: maximum likelihood estimate
PDE: partial differential equation
