# Supplementary material for: The Geographic Spread of the CCR5 Δ32 HIV-Resistance Allele
Source: PLoS Biol. 2005 Oct 18;3(11):e339. doi: 10.1371/journal.pbio.0030339 (PMC1255740; doi:10.1371/journal.pbio.0030339)
Supplement: Supplementary file 1 [file pbio.0030339.sg001.pdf]

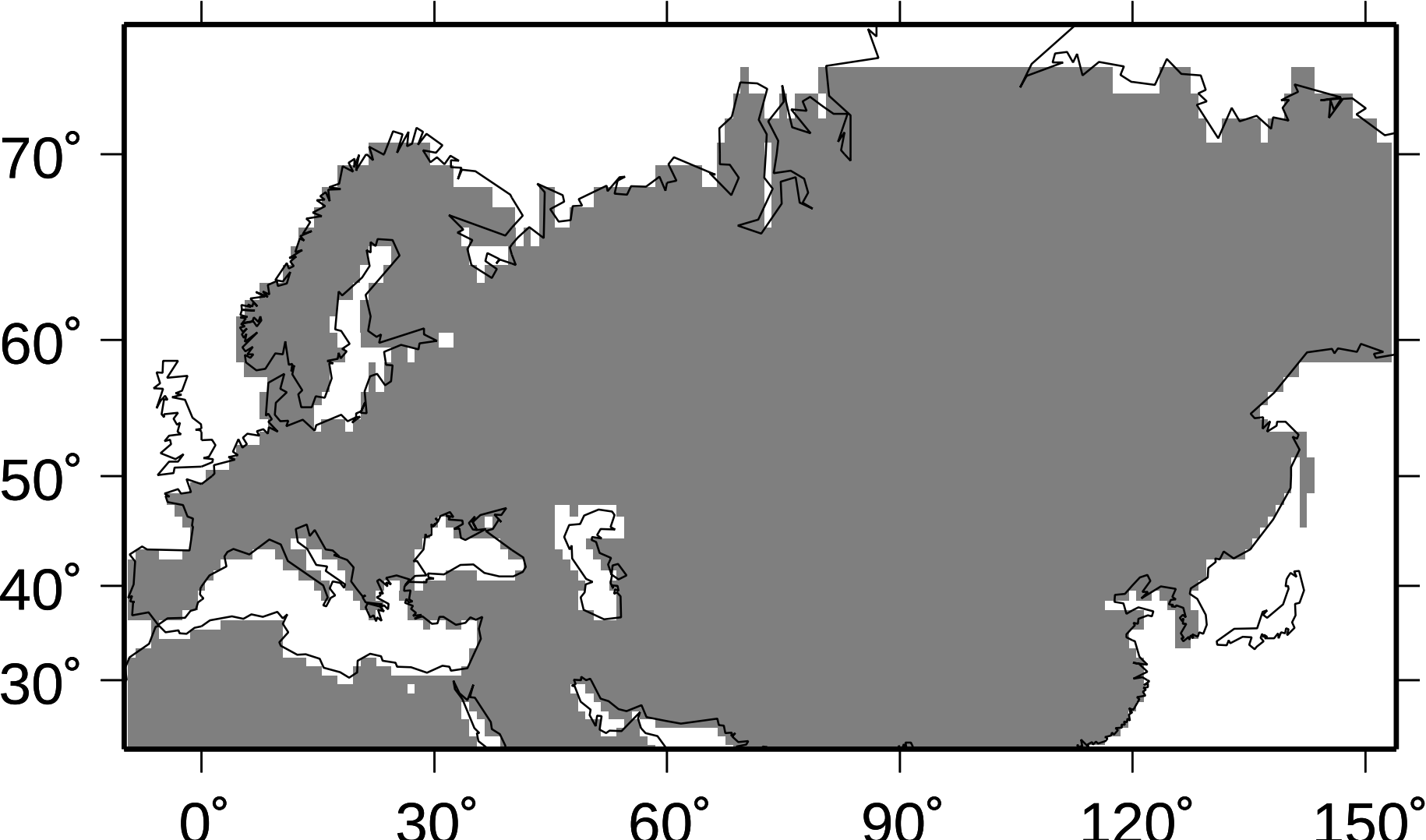

70°
60°
50°
40°
30°
0°
30°
60°
90°
120°
150°
