# Supplementary material for: The Geographic Spread of the CCR5 Δ32 HIV-Resistance Allele
Source: PLoS Biol. 2005 Oct 18;3(11):e339. doi: 10.1371/journal.pbio.0030339 (PMC1255740; doi:10.1371/journal.pbio.0030339)
Supplement: Supplementary file 2 [file pbio.0030339.st001.pdf]

Population	Latitude	Longitude	Δ32 Frequency	Sample Size	Reference
Mordvinians from Saransk (Russia)	54	45	0.163	86	Limborska et al. (2002)
Byelorussians (Byelorussia)	54	24	0.16	80	Limborska et al. (2002)
Finns from Helsinki (Finland)	60	25	0.158	98	Limborska et al. (2002)
Tatars from Elabuga (Russia)	56	52	0.156	45	Limborska et al. (2002)
Swedes from Umea (Sweden)	64	19	0.142	204	Limborska et al. (2002)
Russians from Moscow (Russia)	56	38	0.139	50	Limborska et al. (2002)
Bretons from Bretagne (France)	49	1	0.136	107	Limborska et al. (2002)
Estonia-Mainland	59	26	0.13	250	Kalev et al. (2000)
French from Paris (France)	48	2	0.129	294	Limborska et al. (2002)
Russians from Ryazan (Russia)	55	40	0.12	78	Limborska et al. (2002)
Tatars (Russia)	56	49	0.12	40	Limborska et al. (2002)
Leuven Belgium	51	5	0.119	310	Struyf et al. (2000)
Lithuanians from Vilnius (Lithuania)	54	25	0.115	283	Limborska et al. (2002)
Tatars from Almetyevsk (Russia)	55	52	0.115	48	Limborska et al. (2002)
Udmurts from Malo-Purginski district (Russia)	57	53	0.115	52	Limborska et al. (2002)
Population of Nancy and Strasbourg (France)	48	7	0.111	291	Limborska et al. (2002)
Danes from Copenhagen (Denmark)	56	12	0.11	100	Limborska et al. (2002)
Ukrainians from Kiev (Ukraine)	49	24	0.11	83	Limborska et al. (2002)
French from Lille region (France)	49	3	0.109	101	Limborska et al. (2002)
Mulheim Germany	51	7	0.106	99	Lucotte and Mercier (1998b)
Norwegians from Oslo (Norway)	60	10	0.105	100	Limborska et al. (2002)
French from Montpellier (France)	44	3	0.101	99	Limborska et al. (2002)
French from south of France	44	5	0.098	326	Limborska et al. (2002)
Ukrainians from Lvov (Ukraine)	50	30	0.096	88	Limborska et al. (2002)
Mari from Zvenigovski district (Russia)	56	48	0.096	47	Limborska et al. (2002)
Belgians from Brussels (Belgium)	50	4	0.092	704	Limborska et al. (2002)
Ukrainians from Lugansk (Ukraine)	48	39	0.091	86	Limborska et al. (2002)

Data Table Continued

Population	Latitude	Longitude	Δ32 Frequency	Sample Size	Reference
French from Champagne-Ardenne (France)	47	5	0.087	276	Limborska et al. (2002)
Italians in Milan (Italy)	45	9	0.087	98	Limborska et al. (2002)
Basques (Spain)	44	4	0.086	29	Limborska et al. (2002)
Hungarians from Budapest (Hungary)	47	18	0.086	99	Limborska et al. (2002)
Bern Switzerland	47	7	0.085	64	Lucotte and Mercier (1998b)
Uzbekistan	41	69	0.085	40	Limborska et al. (2002)
Swedish Saamis from Lapland (Sweden)	67	23	0.083	120	Limborska et al. (2002)
Catalonia (Spain)	36	4	0.082	49	Limborska et al. (2002)
Moldavians from Kishinev (Moldavia)	47	29	0.08	56	Limborska et al. (2002)
Barcelona Spain	42	1	0.071	98	Lucotte and Mercier (1998b)
Catalans from Perpignan region (France)	43	2	0.069	102	Limborska et al. (2002)
Portugese from Lisbon (Portugal)	38	-9	0.064	101	Limborska et al. (2002)
Daghestan (Russia)	43	47	0.064	110	Limborska et al. (2002)
Turks from Ankara (Turkey)	39	32	0.063	104	Limborska et al. (2002)
Basques from Bilbao (Spain)	43	-3	0.062	89	Limborska et al. (2002)
Komi Ziryanes from Sisolski district (Russia)	61	50	0.06	50	Limborska et al. (2002)
Kazakstan	43	77	0.06	50	Limborska et al. (2002)
Mordvinians from Staroshayginski district (Russia)	54	44	0.059	51	Limborska et al. (2002)
Italy (Rome)	42	13	0.055	91	Limborska et al. (2002)
Porto Portugal	41	-9	0.052	124	Lucotte and Mercier (1998b)
Nice France	44	6	0.052	124	Lucotte and Mercier (1998a)
Chuvashes from Morgaushevski district (Russia)	56	47	0.051	79	Limborska et al. (2002)
Bashkirs from Sterlibashevski district (Russia)	56	55	0.051	49	Limborska et al. (2002)
Crimea Tatars from Crimea (Ukraine)	46	34	0.05	90	Limborska et al. (2002)
Azerbaijan	41	49	0.05	40	Limborska et al. (2002)
Bashkirs from Ilishevski district (Russia)	55	54	0.05	50	Limborska et al. (2002)

Data Table Continued

Population	Latitude	Longitude	$\Delta 32$ Frequency	Sample Size	Reference
Padua Italy	45	11	0.047	371	Zamarchi et al. (1999)
Athens Greece	39	23	0.042	143	Magierowska et al. (1998)
Uigur (Russia)	51	90	0.04	50	Limborska et al. (2002)
Tuva republic (Russia)	52	95	0.03	50	Limborska et al. (2002)
Pakistan	30	70	0.029	34	Limborska et al. (2002)
Bashkirs from Abselilovski district (Russia)	53	59	0.025	60	Limborska et al. (2002)
Iran	32	53	0.024	84	Voevodin et al. (1998)
Bashkirs from Arkhangelski district (Russia)	54	57	0.022	46	Limborska et al. (2002)
Saudi Arabia	25	41	0.021	241	Limborska et al. (2002)
Basques from Biarritz (France)	43	0	0.018	111	Limborska et al. (2002)
Sindh	26	69	0.017	29	Limborska et al. (2002)
Morocco	32	-5	0.015	167	Elharti et al. (2000)
Punjab	32	73	0.015	34	Limborska et al. (2002)
Syria	35	38	0.014	106	Voevodin et al. (1998)
Tunisia	34	9	0.01	145	Barbouche et al. (2001)
Georgia	42	45	0.0	50	Limborska et al. (2002)
Bengal	24	90	0.0	25	Limborska et al. (2002)
Mongolia	48	107	0.0	59	Limborska et al. (2002)

References

Alvarez V, Lopez-Larrea C, Coto E (1998). Mutational analysis of the CCR5 and CXCR4 genes (HIV-1 co-receptors) in resistance to HIV-1 infection and AIDS development among intravenous drug users. Human genetics 102(4): 483–486.

Barbouche R, Hong L, Dellagi K, Kostrikis L (2001). Contrasting frequencies of CCR5-Delta32 and CCR2-64i allele in Tunisian populations. JAIDS 26: 298–299.

Elharti E, Elaouad R, Simons MJ, Messouak-Elhachimi Z, Gluckman JC, et al. (2000). Frequency of the CCR5 delta 32 allele in the Moroccan population. AIDS Research and Human Retroviruses 16(1): 87–89.

Kalev I, Mikelsaar AV, Beckman L, Tasa G, Parlist P (2000). High frequency of the HIV-1 protective CCR5 delta 32 deletion in native Estonians. European journal of epidemiology 16(12): 1107–1109.

Limborska SA, Balanovsky OP, Balanovskaya EV, Slominsky PA, Schadrina MI, et al. (2002). Analysis of CCR5 delta 32 geographic distribution and its correlation with some climatic and geographic factors. Human heredity 53(1): 49–54.

Lucotte G, Mercier G (1998a). Delta 32 mutation frequencies of the CCR5 coreceptor in different French regions. Comptes Rendus De L Academie Des Sciences Serie I Sciences De La Vie Life Sciences 321(5): 409–413.

Lucotte G, Mercier G (1998b). Distribution of the CCR5 gene 32-bp deletion in Europe. Journal of Acquired Immune Deficiency Syndromes and Human Retrovirology 19(2): 174–177.

Magierowska M, Lepage V, Boubnova L, Carcassi C, de Juan D, et al. (1998). Distribution of the CCR5 gene 32 base pair deletion and sdf1-3 ' a variant in healthy individuals from different populations. Immunogenetics 48(6): 417–419.

Ruiz A, Royo J, Rubio A, Borrego S, Leal M, et al. (2001). Spectrofluorimetric analysis of CCR5-Delta 32 allele using real-time polymerase chain reaction: prevalence in southern Spanish HIV+ patients and noninfected population. AIDS Res Hum Retroviruses 17: 191–193.

Struyf F, Thoelen I, Charlier N, Keyaerts E, Van der Donck I, et al. (2000). Prevalence of CCR5 and CCR2 HIV-coreceptor gene polymorphisms in Belgium. Hum Hered 50: 304–307.

Voevodin A, Samilchuk E, Dashti S (1998). A survey for 32 nucleotide deletion in the CCR-5 chemokine receptor gene (delta CCR-5) conferring resistance to human immunodeficiency virus type 1 in different ethnic groups and in chimpanzees. Journal of medical virology 55(2): 147–151.

Zamarchi R, Indraccolo S, Minuzzo S, Coppola V, Gringeri A, et al. (1999). Frequency of a mutated CCR-5 allele (delta32) among Italian healthy donors and individuals at risk of parenteral HIV infection. AIDS Res Hum Retroviruses 15: 337–344.
